# Lead-Free Halide Double Perovskite Materials: A New Superstar Toward Green and Stable Optoelectronic Applications

**DOI:** 10.1007/s40820-019-0244-6

**Published:** 2019-02-27

**Authors:** Liang Chu, Waqar Ahmad, Wei Liu, Jian Yang, Rui Zhang, Yan Sun, Jianping Yang, Xing’ao Li

**Affiliations:** 1 New Energy Technology Engineering Laboratory of Jiangsu Province & School of Science, Nanjing University of Posts and Telecommunications (NJUPT), Nanjing, 210023 People’s Republic of China; 2 Key Laboratory for Organic Electronics & Information Displays & Institute of Advanced Materials, Jiangsu National Synergistic Innovation Center for Advanced Materials, School of Materials Science and Engineering, Nanjing University of Posts and Telecommunications (NJUPT), Nanjing, 210023 People’s Republic of China; 3 Wuhan National Laboratory for Optoelectronics (WNLO), Huazhong University of Science and Technology (HUST), Wuhan, 430074 People’s Republic of China

**Keywords:** Halide double perovskite, Optoelectronic applications, Efficiency, Stability, Toxicity

## Abstract

Lead-based halide perovskites have emerged as excellent semiconductors for a broad range of optoelectronic applications, such as photovoltaics, lighting, lasing and photon detection. However, toxicity of lead and poor stability still represent significant challenges. Fortunately, halide double perovskite materials with formula of A_2_M(I)M(III)X_6_ or A_2_M(IV)X_6_ could be potentially regarded as stable and green alternatives for optoelectronic applications, where two divalent lead ions are substituted by combining one monovalent and one trivalent ions, or one tetravalent ion. Here, the article provides an up-to-date review on the developments of halide double perovskite materials and their related optoelectronic applications including photodetectors, X-ray detectors, photocatalyst, light-emitting diodes and solar cells. The synthesized halide double perovskite materials exhibit exceptional stability, and a few possess superior optoelectronic properties. However, the number of synthesized halide double perovskites is limited, and more limited materials have been developed for optoelectronic applications to date. In addition, the band structures and carrier transport properties of the materials are still not desired, and the films still manifest low quality for photovoltaic applications. Therefore, we propose that continuing efforts are needed to develop more halide double perovskites, modulate the properties and grow high-quality films, with the aim of opening the wild practical applications.

## Highlights

- Lead-based halide perovskite materials have revealed excellent properties in optoelectronic applications. However, the material stability and the toxicity of lead still hinder their large-scale commercial applications.
- Lead-free halide double perovskite materials possess the characteristics of environmental friendliness, exceptional stability and tunable optoelectronic properties.
- A limited number of halide double perovskites have been synthesized, and extremely few have been developed for optoelectronic applications. Continuing effort is needed to explore more halide double perovskites and modulate the properties for their further applications.

## Graphical abstract

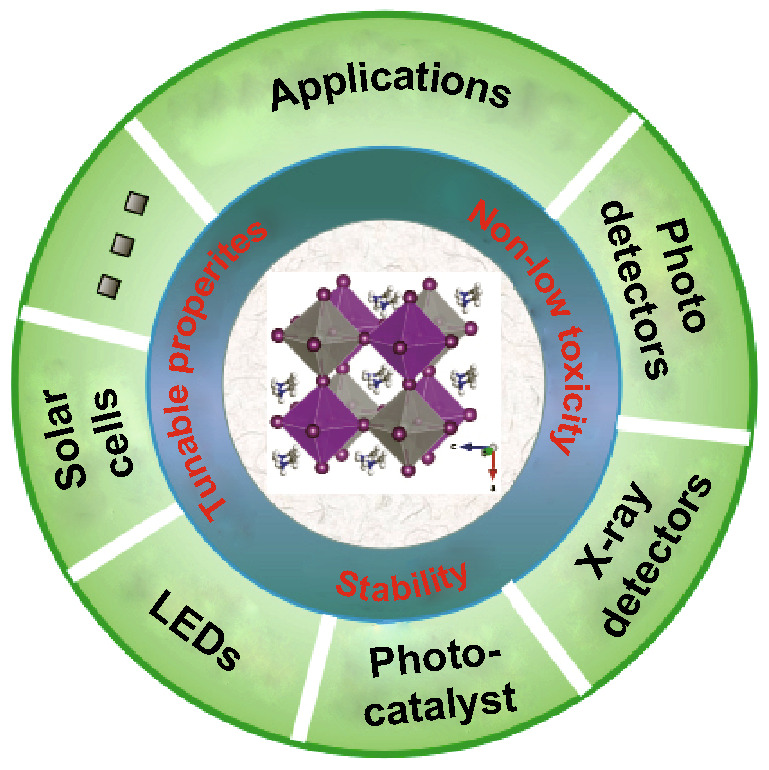

## Introduction

Halide perovskites with the generic formula AM(II)X_3_ (A: CH_3_NH_3_^+^, CH(NH_2_)_2_^+^, Cs^+^; M(II): Pb^2+^, Sn^2+^; X: I^−^, Br^−^, Cl^−^) can be divided into two broad categories according to the A-location cation: organic–inorganic hybrid and all-inorganic halide perovskites. Initially, halide perovskites of CH_3_NH_3_PbX_3_ (X=Br, I) were utilized as light sensitizer to replace dyes in dye-sensitized solar cells with liquid electrolyte in 2009 [1]. However, due to the underdeveloped efficiency ~ 3.8% and stability, the initial perovskite solar cells (PSCs) did not capture widespread attention. Until 2012, a solid hole transport layer was substituted for the liquid electrolyte to develop all-solid-state PSCs. Most strikingly, the efficiency and stability were simultaneously distinctly improved [2], which stimulated “perovskite fever” [3–5]. Recently, the certified efficiency of PSCs has risen to 23.7%, which is comparable to conventional silicon solar cells [6]. The rocketing improvement of efficiency is attributed to the suitable direct bandgap, strong absorption coefficient, long-range charge diffusion length, balanced electron–hole mobility, high dielectric constant, excellent carrier mobility and small exciton binding energy of halide perovskites [7–10]. Besides solar cells, these materials have been applied in other optoelectronic applications, such as light-emitting diodes (LEDs) [11, 12], lasers [13], photodetectors [14, 15] and X-ray detectors [16].

Although efficiency of PSCs has been progressively grown, there are still huge barriers which limit the commercial applications. For instance, the long-term stability over 10 years is the most critical obstacle to restrict the commercialization [17]. Currently, the PSCs cannot remain stable more than 1 year outdoors, whereas silicon solar cells are usually guaranteed to work for at least 25 years. Moisture, temperature, oxygen and extreme light levels all cause PSCs to decompose. Moisture is the worst problem because reaction with the water forms hydrates to destroy the crystal structures [18]. To improve the stability of PSCs, six main solutions have been adopted: (1) regulating the crystal structures to improve phase stability by doping [19], (2) reducing crystal defects to limit penetrating channels from external environment [20, 21], (3) designing new stable halide perovskite materials [22, 23], (4) using stable inorganic charge transport layers [24], (5) adopting 2D halide perovskite materials [25, 26] and (6) packaging the devices [27]. Based on the above solutions, cell lifetimes have extended from a few days to months. Nevertheless, there is still a long way to go toward long-time stability over tens of years. Also, toxic lead is still necessary to achieve high performance. Lead pollution will do serious harm to human health, such as fatigue, muscle weakness, clumsiness and clouded consciousness [28]. Extensive efforts have been paid to design new non-/low-toxic and stable halide perovskites for solar cells as well as other optoelectronic applications [29–31]. Sn^2+^ and Ge^2+^ ions could be expected to replace Pb^2+^ ions in perovskites. However, the Sn^2+^ and Ge^2+^ cations tend to undergo oxidation due to the high-energy-lying 5 s orbitals, rendering the corresponding perovskite extremely unstable in ambient atmosphere [30, 31]. Similarly, Bi^3+^ and Sb^3+^ ions with the similar isoelectronic structure have been substituted for Pb^2+^ ions to develop stable and lead-free perovskites. However, inherently low-dimensional structures of the A_3_M(III)_2_X_9_ perovskites result in less impressive performance [22, 23]. Afterward, doping bivalent metal ions in moderation not only decreases lead content, but also enhances the efficiency and stability, such as Sr^2+^ [32], Co^2+^ [33] and Zn^2+^ [34]. The main reason is that the metal ion-based doping can moderately improve the quality of perovskite films. Simultaneously, doping a certain type of heterovalent metal ions can engineer the band structure of perovskite to enhance the performance, such as Ag^+^ [35], Sb^3+^ [36] and Bi^3+^ [37]. However, the doping method cannot thoroughly solve any issues of stability or toxicity.

**Fig. 1:**
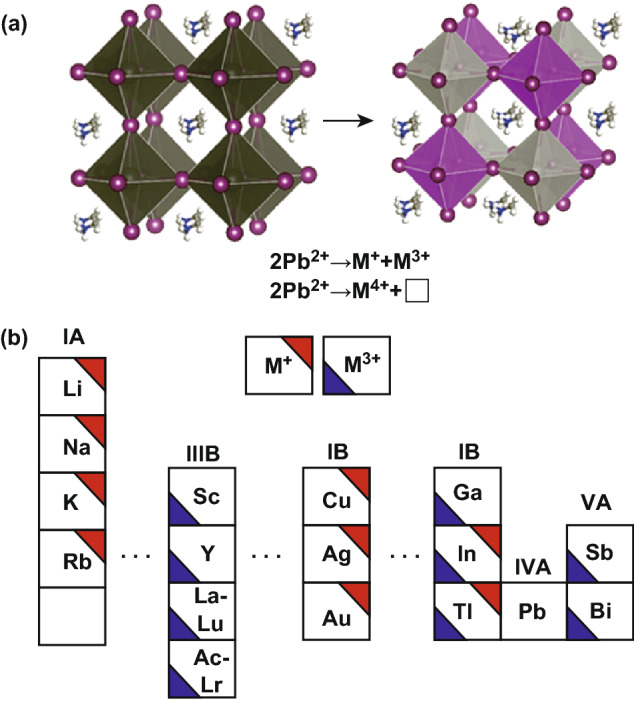
**a** Schematic illustration of the transformation from APbX_3_ to A_2_M(I)M(III)X_6_ or A_2_M(IV)X_6_, where two toxic Pb^2+^ ions are substituted by combining M^+^ and M^3+^ ions (or M^4+^ ion). “□” denotes M-site vacancy. **b** Elements of M-location cations with M^+^ and M^3+^ in the periodic table for halide double perovskites. When any M-site elements are localized at IA or IIIA groups of the periodic table, the materials have direct bandgaps

Recently, halide double perovskites (A_2_M(I)M(III)X_6_, A_2_M(IV)X_6_) have been proposed as stable and green alternatives to lead halide perovskites, where two toxic lead ions are substituted by combining one monovalent and one trivalent ions, or one tetravalent ion and one vacancy site (marked as “□”) to yield the same overall charge balance as the conventional perovskites, as shown in Fig. 1a [38]. The formula of A_2_M(IV)X_6_ is considered as vacancy-ordered halide double perovskites, which is the analogy of A_2_M(I)M(III)X_6_. Because of the cubic structure $({\text{F}}\overline{{{\text{m}}3}}{\text m})$ to extend three dimensions with corner-sharing metal halide octahedra, halide double perovskites have attracted extensive attention as promising optoelectronic candidates. In this review, we highlight lead-free halide double perovskite materials and their related optoelectronic applications including photodetectors, X-ray detectors, photocatalyst, LEDs and solar cells. The synthesized halide double perovskites exhibited pleasurable stability. But only a limited number of halide double perovskites have been synthesized, and extremely few have been developed for optoelectronic applications. In addition, the band structures and carrier transport properties of the materials are still not desired, and the films still manifest low quality for photovoltaic applications. It is universally acknowledged that significant effort is needed to discover more candidates and modulate the properties for their further applications.

## Halide Double Perovskite Materials

Besides stability, the band structures and carrier transport properties of the materials predetermine to a large extent the specific applications and their performance. In halide perovskites with the chemical formula APbX_3_, the valence and conduction bands are predominantly made up of the Pb-6p and X-5p orbitals. In addition, the size of the A-location cation affects the PbX_6_ octahedra to distort/tilt, which can slightly modify the band structures [39]. Similarly, the band structures of the halide double perovskites are mainly decided by the M(I)-, M(III)- (or M(IV)-) and X-site atoms. The elements of M-location cations with monovalent and trivalent in the periodic table toward halide double perovskites are shown in Fig. 1b. The elements in IA, IB and IIIA groups can occupy the M^+^ sites, and the elements in IIB, IIIA and VA groups are found at the M^3+^ sites. When any M-site elements of the halide double perovskites are localized at IA or IIIA groups of the periodic table, the materials have direct bandgaps. The In and Tl elements in IIIA group possess valencies +1 and +3. The vacancy-ordered halide double perovskites (A_2_M(IV)X_6_) all have direct bandgaps. Theoretically, there are a lot of halide double perovskites, though the exact formula must be considered two aspects of the Goldschmidt’s rule and thermodynamic stability [40]. However, only a limited number of halide double perovskites have been synthesized so far, as listed in Table 1 including their synthetic methods, where MA is CH_3_NH_3_.

**Table 1:** Summary of the prepared halide double perovskites

Materials	Morphology	Synthetic method	References
Cs_2_NaMCl_6_ (M=Am, Bk, Tl, Bi, Lu, etc.)	Powder or single crystal	Evaporating HCl solution to dryness, or heating anhydrous chlorides, or precipitating from cold HCl solution, or growing crystals from dilute HCl solution by cooling evaporation	[41–47]
Cs_2_AgBiCl_6_	Powder	Melt crystallization Precipitation from heated acid solution	[48, 49]
	Nanocrystal	Hot injection Antisolvent recrystallization	[50, 51]
Cs_2_AgBiBr_6_	Powder	Melt crystallization Precipitation from heated acid solution	[48]
	Single crystal	Cooling crystallization	[52, 54, 55]
	Nanocrystal	Hot injection Antisolvent recrystallization	[50, 51, 56]
Cs_2_(Ag_1−*a*_Bi_1−*b*_)Tl_*x*_Br_6_	Single crystal	Cooling crystallization	[57]
Cs_2_Ag(Bi_1−*x*_M_*x*_)Br_6_ (M=In, Sb)	Single crystal	Melt crystallization	[58]
Cs_2_AgBiI_6_	Nanocrystal	Antisolvent recrystallization Anion exchange	[50, 51]
Cs_2_AgInCl_6_	Single crystal Powder	Cooling crystallization Precipitating from cold HCl solution	[59–61]
Mn-doped Cs_2_AgInCl_6_	Microcrystal	Hot injection	[62]
Cs_2_NaBiI_6_	Microcrystal	Hydrothermal method	[64]
Cs_2_SnI_6_	Microcrystal Nanocrystal	Hot injection	[74–76]
Bi-doped Cs_2_SnCl_6_	Single crystal	Cooling crystallization	[77]
Cs_2_PdBr_6_	Single crystal	Cooling crystallization	[78]
	Nanocrystal	Antisolvent recrystallization	[79]
Cs_2_PdI_6_	Nanocrystal	Anion exchange	[79]
A_2_TiBr_6_ (A=K, Rb, Cs)	Powder	Molten salt	[80]
Cs_2_TiI_*x*_Br_6−*x*_ (*x* = 0, 2, 4, 6)	Powder	Melt crystallization	[81]
(MA)_2_KBiCl_6_	Powder	Evaporating HCl solution to dryness	[38]
(MA)_2_TlBiBr_6_	Single crystal	Hydrothermal method	[82]
(MA)_2_AgBiBr_6_	Powder	Evaporating HBr solution to dryness	[83]
(MA)_2_AgSbI_6_	Powder	Melt crystallization	[84]
(MA)_2_AgBiI_6_	Powder	Melt crystallization	[85]
(MA)_2_KGdCl_6_ (MA)_2_KYCl_6_	Powder	Evaporating HCl solution to dryness	[86]
(MA)_2_AgInBr_6_	Single crystal	MAPbBr_3_-induced crystallization	[87]
(MA)_2_SnI_6_	Powder	Mixed iodides	[88]

### All-Inorganic Halide Double Perovskites

Initially, all-inorganic halide double perovskite of Cs_2_NaAmCl_6_ was prepared in 1968 by evaporating HCl solution containing cations to dryness [41]. Afterward, a few all-inorganic halide double perovskites (Cs_2_NaM(III)Cl_6_) were fabricated by the same method [42–44]. In those days, ferroelectric phase transition was considered particularly attracted for all-inorganic halide double perovskites [45–47], which was observed on the cooling Cs_2_NaBiCl_6_ [47].

**Fig. 2:**
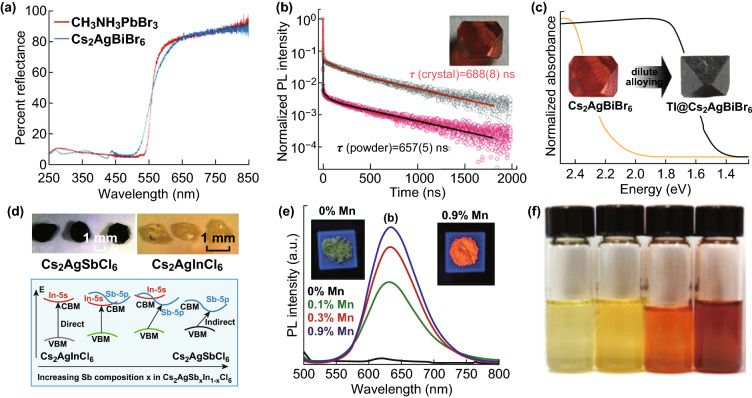
**a** Diffuse reflectance spectra of Cs_2_AgBiBr_6_ and CH_3_NH_3_PbBr_3_. Reproduced with permission from Ref. [48]. **b** Time-resolved room-temperature PL and fits for the PL decay time (τ) in powder and single-crystal samples. The inset is the photograph of a Cs_2_SgBiBr_6_ single crystal. Reproduced with permission from Ref. [52]. **c** Apparent bandgaps of Cs_2_AgBiBr_6_ and Cs_2_(Ag_1−a_Bi_1−b_)TlxBr_6_ (*x* = *a* + *b* = 0.075) single crystals extracted by linear fits to α^2^ vs. E (direct gap) and α^1/2^ vs. E plots (indirect gap). Reproduced with permission from Ref. [57]. **d** Photographs of Cs_2_AgSbCl_6_ and Cs_2_AgInCl_6_ single crystals (top), and band diagram. A change in the character of the conduction band minimum (CBM) from s orbital derived to p orbital derived while having the valence band maximum (VBM) primarily Ag-d states results in a transition from direct to indirect bandgap. Reproduced with permission from Ref. [59]. **e** PL spectra of Mn-doped Cs_2_AgInCl_6_ with different Mn contents, after excitation with 340 nm light. Insets show photographs of luminescence from powder samples under UV light. Reproduced with permission from Ref. [62]. **f** Photograph of dilute toluene solutions of Cs_2_AgBiBr_6_, Cs_2_AgBiBr_5.2_I_0.8_, Cs_2_AgBiBr_1.6_I_4.4_ and Cs_2_AgBiI_6_ nanocrystals. Reproduced with permission from Ref. [51]

In light of the obsessive interest in halide perovskites, halide double perovskites have attained great concern in recent 2 years. In 2016, three different groups almost simultaneously reported Cs_2_AgBiX_6_ (X=Cl or Br) as a promising alternative to the lead halide perovskites, which crystallizes in cubic ${\text{F}}\overline{{{\text{m}}3}}{\text m}$ symmetry and shows light absorption at the visible range of the spectrum [48, 49, 52]. Woodward et al. [48] measured diffuse reflectance to reveal bandgaps of 2.19 and 2.77 eV for Cs_2_AgBiBr_6_ and Cs_2_AgBiCl_6_, respectively, as shown in Fig. 2a. The band structure calculation indicates that the interaction between Ag 4d orbitals and 3p/4p orbitals of the halide ions modifies valence band, leading to an indirect bandgap. Both compounds are stable when exposed to air; however, Cs_2_AgBiBr_6_ degrades over a period of weeks when exposed to ambient air and light. Meanwhile, Karunadasa et al. [52] tried to synthesize highly thermal and moisture-stable Cs_2_AgBiBr_6_ single crystal with an indirect bandgap of 1.95 eV and photoluminescence (PL) lifetime of ~ 660 ns, as shown in Fig. 2b, which is very encouraging for photovoltaic applications. Then, Giustino et al. [49] designed and synthesized Cs_2_AgBiCl_6_ with bandgaps between 1.95 and 3.04 eV. However, the bandgap of Cs_2_AgBiX_6_ (X=Cl, Br) is indirect and slightly large, not ideal for thin-film photovoltaic applications. To engineer the bandgaps, Karunadasa et al. [52, 57] tried to incorporate Tl as a dilute impurity into their reported Cs_2_AgBiBr_6_ single crystals. After incorporating Tl, the color changed to opaque black from translucent orange, which reflects the reduction of the bandgaps, as shown in Fig. 2c. The Tl content can be tuned across the series Cs_2_(Ag_1−*a*_Bi_1−*b*_)Tl_x_Br_6_ (0.003 < *x* = *a* + *b* < 0.075), and Cs_2_(Ag_1−*a*_Bi_1−*b*_)Tl_x_Br_6_ (*x* = 0.075) displays low indirect and direct bandgaps of 1.40 and 1.57 eV, respectively. Importantly, time-resolved photoconductivity measurements reveal long-lived carriers with microsecond lifetimes in the alloyed material, suggesting that carriers can be efficiently extracted in a solar cell [53]. The alloyed perovskite is the first halide double perovskite to show comparable bandgap and carrier lifetime to those of CH_3_NH_3_PbI_3_, but unfortunately the content of Tl is still toxic [53, 57]. In 2017, Yan et al. [58] used Cs_2_AgBiBr_6_ as a host to engineer the bandgap through alloying of In^3+^ and Sb^3+^. Cs_2_Ag(Bi_1−*x*_M_*x*_)Br_6_ (M=In, Sb) accommodates up to 75% In^3+^ with increased bandgap, and up to 37.5% Sb^3+^ with reduced bandgap, that is, enabling ~ 0.41 eV bandgap modulation through introduction of the two metals, with smallest value of 1.86 eV for Cs_2_Ag(Bi_0.625_Sb_0.375_)Br_6_. Band structure calculations indicate that opposite bandgap shift directions associated with Sb/In substitution arise from different atomic configurations for these atoms. Similarly, McQueen et al. [59] designed indirect and direct bandgap transitions using alloy strategy, as shown in Fig. 2d. The synthesized Cs_2_AgSbCl_6_ and Cs_2_AgInCl_6_ single crystals have indirect and direct bandgaps, respectively. When increasing Sb composition *x* in Cs_2_AgSb_*x*_In_1−*x*_Cl_6_, the compounds gradually transit from direct to indirect bandgap. Later on, Giustino et al. [60] identified that Cs_2_InAgCl_6_ has direct bandgap of 3.3 eV and the compound is found to be photosensitive and turns reversibly from white to orange under ultraviolet (UV) illumination. Typically, Cs_2_InAgCl_6_ exhibits a wide bandgap which limits its application in the visible region. In 2018, Nag et al. [62] imparted the visible-light emission property in direct bandgap Cs_2_AgInCl_6_ by doping Mn^2+^ ions, as shown in Fig. 2e. Cs_2_AgInCl_6_ host absorbs UV light and then transfers the excitation energy to Mn d electrons. For X-site in perovskites, the Bohr radii gradually increase from F to I elements, resulting in increasing tightly bound nature and bandgaps [63]. Obviously, the X elements in the synthesized halide double perovskites are usually Cl or Br, and the bandgaps are relatively large. Gamelin et al. [51] synthesized Cs_2_AgBiX_6_ (X=Cl, Br) colloidal nanocrystals by a hot-injection approach, which were converted to new materials including Cs_2_AgBiI_6_ with a narrow bandgap about 1.75 eV through anion exchange. Figure 2f shows the photograph of dilute toluene solutions of (left to right) Cs_2_AgBiBr_6_, Cs_2_AgBiBr_5.2_I_0.8_, Cs_2_AgBiBr_1.6_I_4.4_ and Cs_2_AgBiI_6_ nanocrystals, and the dark red color of Cs_2_AgBiI_6_ reflects that the absorption extends throughout the visible region. Recently, Ma et al. [64] reported first time the synthesis of novel halide double perovskite material of Cs_2_NaBiI_6_ and determined its crystal structure by XRD and XPS tests and optical properties by UV–Vis absorption spectra. Cs_2_NaBiI_6_ has a low direct bandgap of 1.66 eV and exhibits high stability against moisture and oxygen in ambient air.

To seek halide double perovskites theoretically, Zhang et al. [65–69] designed a series of all-inorganic halide double perovskites through first-principles calculations for the last 2 years. Photovoltaic-functionality-directed material screening process involves totally sixty-four candidate materials to identify 11 Sb- and Bi-based optimal materials containing intrinsic thermodynamic stability, suitable bandgaps, small carrier effective masses and low excitons binding energies as promising photovoltaic materials. When the monovalent ion is Tl^+^ or In^+^, the materials have direct bandgap, among which Cs_2_InSbCl_6_ and Cs_2_InBiCl_6_ have the bandgap about 1.0 eV, and show the theoretical maximum performance comparable to that of CH_3_NH_3_PbI_3_. However, the Tl is still toxic and the In^+^ is unstable by spontaneous oxidation into In^3+^ [70]. They simultaneously designed trivalent In^3+^ ion with monovalent Ag^+^ or Cu^+^ ion to find halide double perovskites [69]. Among them, Rb_2_CuInCl_6_, Rb_2_AgInBr_6_ and Cs_2_AgInBr_6_ have direct bandgaps of 1.36, 1.46 and 1.50 eV, respectively, and theoretical spectroscopic limited maximal efficiency comparable to CH_3_NH_3_PbI_3_.

It is noteworthy that there is another type of halide double perovskite as vacancy-ordered A_2_M(IV)X_6_ (M(IV)=Sn, Ti, Pd, Te, etc.), which not only proposes direct bandgaps, but also intrinsic stability and non-/low toxicity [71–73]. In 2014, Kanatzidis et al. [74] prepared Cs_2_SnI_6_ microcrystal by a hot-injection method. Cs_2_SnI_6_ nanocrystal was prepared with controlled shapes by the same method [75, 76]. The bandgap of the Cs_2_SnI_6_ was found to be varied in the range of 1.36–1.67 eV. Quantum confinement effect has been observed for the nanoparticles of dimension below 8 nm. In 2018, Tang et al. [77] fabricated Bi-doped Cs_2_SnCl_6_ single crystals, where the photoluminescence was observed from Bi^3+^ ions. In 2017, Snaith et al. [78] reported Cs_2_PdBr_6_ single crystal, which exhibits long-lived photoluminescence, direct bandgap of 1.6 eV and long-term stability. In 2018, Kuang et al. [79] used antisolvent recrystallization method to prepare Cs_2_PdBr_6_ nanocrystals with an average particle diameter of 2.8 nm. Such Cs_2_PdBr_6_ nanocrystals not only display high stability against light illumination (one sun for more than 1000 h), moisture (70% for 2 months) and high temperature (120 °C for 600 h) conditions but also possess intriguing optical and ultrafast photophysical properties with a narrow direct bandgap of 1.69 eV. Further, a fast anion exchange method is adopted to prepare the Cs_2_PdI_6_ nanocrystals. Another Ti-based vacancy-ordered halide double perovskites can start back in the early 1960s, and K_2_TiBr_6_, Rb_2_TiBr_6_ and Cs_2_TiBr_6_ were prepared by using fused SbBr_3_ as the solvent [80]. However, the studies of them in optoelectronics have not been performed until 2018, and Zhou et al. [81] synthesized a series of Cs_2_TiI_*x*_Br_6−*x*_ (*x* = 0, 2, 4, 6) powers using the melt crystallization method, of which the bandgap can be tuned continuously from 1.02 to 1.78 eV. The present vacancy-ordered halide double perovskites possess several desirable properties, including ultra-stability, suitable direct bandgap, excellent optical absorption and benign defect.

### Hybrid Halide Double Perovskites

**Fig. 3:**
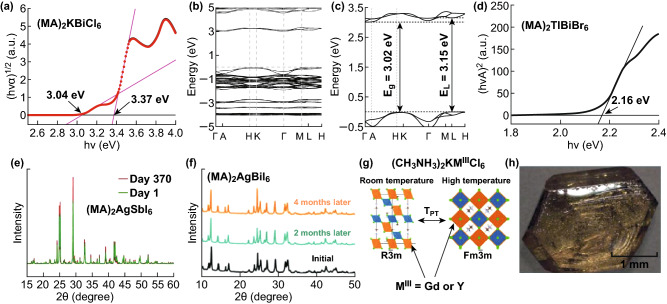
**a** Tauc plot (assumed indirect bandgap), **b-c** calculated band structure of (MA)_2_KBiCl_6_ and the enlarged view of the band structure near the bandgap. Reproduced with permission from Ref. [38]. **d** Tauc plot (assumed direct bandgap) of (MA)_2_TlBiBr_6_. Reproduced with permission from Ref. [82]. **e** XRD patterns of fresh (MA)_2_AgSbI_6_ compared to (MA)_2_AgSbI_6_ after 370 days of exposure to air. Reproduced with permission from Ref. [84]. **f** Air stability of (MA)_2_AgBiI_6_. Reproduced with permission from Ref. [85]. **g** Schematic illustration of rhombohedral-to-cubic phase transition on high-temperature heating for (MA)_2_KGdCl_6_ and (MA)_2_KYCl_6_. Reproduced with permission from Ref [86]. **h** Photograph of a (MA)_2_AgBiBr_6_ single crystal. Reproduced with permission from Ref. [87]

To date, there have been synthesized nine hybrid halide double perovskites to the best of our knowledge, which are (MA)_2_KBiCl_6_ [38], (MA)_2_TlBiBr_6_ [82], (MA)_2_AgBiBr_6_ [83], (MA)_2_AgSbI_6_ [84], (MA)_2_AgBiI_6_ [85], (MA)_2_KGdCl_6_ [86], (MA)_2_KYCl_6_ [86], (MA)_2_AgInBr_6_ [87], and (MA)_2_SnI_6_ [88], as listed in Table 1. In 2016, the first hybrid halide double perovskite (MA)_2_KBiCl_6_ was synthesized by evaporating HCl solution to dryness [38]. In the Tauc plot from the reflectance spectrum, there are two edges with values of 3.04 and 3.37 eV in Fig. 3a, in accordance with the theoretically calculated indirect bandgap of 3.02 eV and direct bandgap of 3.15 eV as shown in Fig. 3b, c. Further, density functional theory screening of (MA)_2_MBiX_6_ (M=K, Cu, Ag, Tl; X=Cl, Br, I) shows that systems with bandgaps similar to those of the CH_3_NH_3_PbX_3_ lead compounds can be expected for M=Cu, Ag, Tl. Motivated by these findings, (MA)_2_TlBiBr_6_, isoelectronic with CH_3_NH_3_PbBr_3_, was synthesized and found to have a direct bandgap of 2.16 eV, as shown in Fig. 3d [82]. However, despite its interesting electronic properties, the severe toxicity of Tl precludes (MA)_2_TlBiBr_6_ from being a practical alternative to the Pb analog. Subsequently, they synthesized a hybrid halide double perovskite, (MA)_2_AgBiBr_6_, that has a low indirect bandgap of 2.02 eV and is relatively stable and nontoxic [83]. The material is stable in air and moisture and exhibits higher decomposition temperature than that of MAPbBr_3_. In 2017, the same group synthesized (MA)_2_AgSbI_6_ [84] and (MA)_2_AgBiI_6_ [85] with indirect bandgap of ~ 2 eV and exhibited high stability in air, as the XRD patterns shown in Fig. 3e, f. Though Sb and Bi are in the same group, there are still some differences between them. Compared with Bi atom, Sb atom has a smaller mass and ion radius and the smaller ion radius of Sb leads to structural distortion of (MA)_2_AgSbI_6_, while the structure of (MA)_2_AgBiI_6_ is the orthogonal phase. Besides, due to the seriously relativistic effects in heavy metal atom Bi, the impact of spin–orbit coupling on bandgap of (MA)_2_AgBiI_6_ is more pronounced. Simultaneously, (MA)_2_KGdCl_6_ and (MA)_2_KYCl_6_ have been synthesized by a solution evaporation method, which adopt a rhombohedral structure with R͞3 m symmetry [86]. Both phases exhibit a rhombohedral-to-cubic phase transition on heating to ~ 435 K as shown in Fig. 3g. Density functional calculations on the rhombohedral phase indicate that both materials have large direct bandgaps about 5 eV and mechanical stability. In 2018, (MA)_2_AgInBr_6_ single crystal [87], as shown in Fig. 3h, is obtained through the use of Pb^2+^ (from CH_3_NH_3_PbBr_3_) to modulate the soluble intermediates and force the formation. In the same year, (MA)_2_SnI_6_ powder was obtained by mixing SnI_4_ with CH_3_NH_3_I powder at room temperature [88]. The powder was evaporated in a tungsten boat at 120 °C in a vacuum chamber to deposit the (MA)_2_SnI_6_ films. The films have a direct bandgap of 1.81 eV with a strong absorption coefficient of ~ 7 × 10^4^ cm^−1^. In addition, the films were n type with a carrier concentration of ~ 2 × 10^15^ cm^−3^ and an electron mobility of ~ 3 cm^2^/V/s. Moreover, the conductivity was increased by a factor of 4 under simulated solar illumination (100 mW cm^−2^). These results indicate that (MA)_2_SnI_6_ is a lead-free optical semiconductor suitable for solar cell applications. The synthesized hybrid halide double perovskites indicate substantially better stability, unlike CH_3_NH_3_PbI_3_. However, to the best of our knowledge, there are no reports about applications of hybrid halide double perovskites.

## Optoelectronic Applications

### Photodetectors

**Fig. 4:**
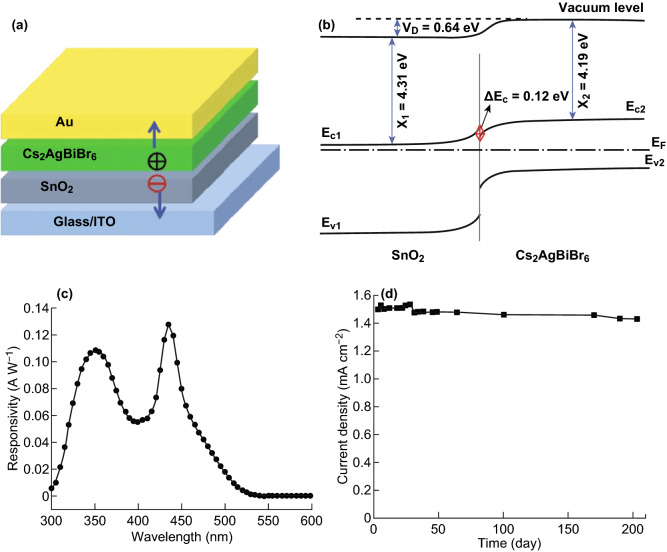
**a** Configuration diagram of Cs_2_AgBiBr_6_-based photodetector. **b** Band scheme diagram of Cs_2_AgBiBr_6_/SnO_2_ heterojunction. **c** Responsivity of photodetector at zero bias. **d** Long-term stability test of Cs_2_AgBiBr_6_-based photodetector. Reproduced with permission from Ref. [91]

In 2017, Tang et al. [61] successfully prepared high-quality Cs_2_AgInCl_6_ single crystals with a low trap state density ((8.6 ± 1.9) × 10^8^ cm^−3^) by a hydrothermal method, which were further applied in UV detectors. The obtained results experimentally verified the existence of parity-forbidden transition [89] and identified that the oxygen was effective on the optical properties. By eliminating oxygen contamination in vacuum, Cs_2_AgInCl_6_ single crystal-based UV detector showed best performance with visible blind, high ON–OFF ratio (~ 500), fast photoresponse (~ 1 ms), low dark current (~ 10 pA at 5 V bias) and high detectivity (~ 1012 Jones). If the Cl is replaced by Br in Cs_2_AgInX_6_ compounds, the bandgaps reduce and absorption range can be extended to visible region. Shi et al. [90] used a one-step spin-coating method to prepare Cs_2_AgBiBr_6_ thin films for photodetectors. The device exhibits high responsivity of 7.01 A W^−1^, ON–OFF ratio of 2.16 × 10^4^, specific detectivity of 5.66 × 10^11^ Jones, EQE of 2146% and demonstrates remarkable stability against the water and oxygen degradation. Wang et al. [91] fabricated highly efficient and stable self-powered UV and deep-blue detector based on Cs_2_AgBiBr_6_/SnO_2_ heterojunction, as shown in Fig. 4a. The photogenerated carriers in Cs_2_AgBiBr_6_ film can be separated at the Cs_2_AgBiBr_6_/SnO_2_ heterojunction interface by its built-in field, as illustrated in Fig. 4b. The device is self-powered with two responsivity peaks at 350 and 435 nm, which is suitable for UV (320–400 nm) and deep-blue light detection, as shown in Fig. 4c. A high responsivity of 0.11 A W^−1^ at 350 nm and a quick response time of less than 3 ms are obtained, which are significantly higher than those of other semiconductor oxide heterojunction-based UV detectors. More importantly, the photocurrent shows no noticeable degradation after more than 6 months of storage in ambient conditions without encapsulation, as shown in Fig. 4d. In addition, the aforementioned vacancy-ordered double perovskites of Cs_2_PdBr_6_ single crystal and Cs_2_SnI_6_ nanocrystal were successfully applied in stable and fast photodetectors [75, 78]. Consequently, the halide double perovskite-based photodetectors displayed high efficiency and stability and environmental friendliness, which are potential alternatives for practical applications.

### X-ray Detectors

**Fig. 5:**
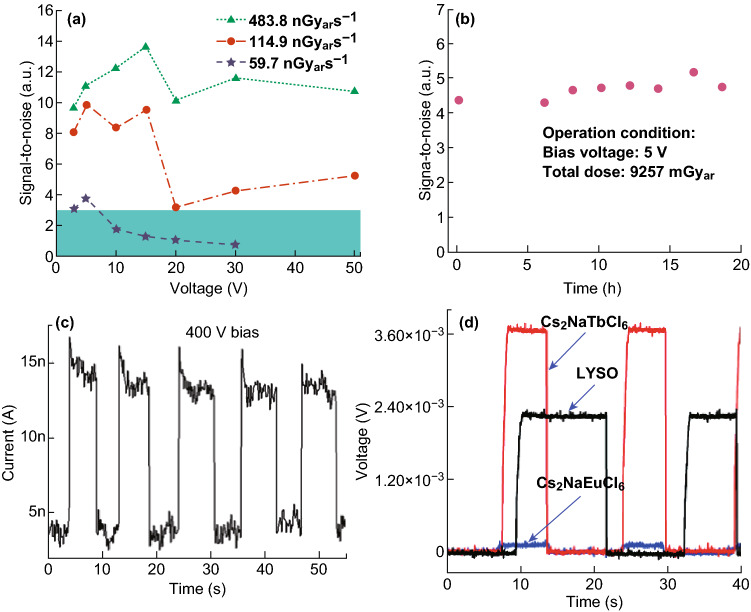
**a** Signal-to-noise ratio of the device derived by calculating the standard deviation of the X-ray photocurrent. The red dashed line represents a SNR of 3, and thus the detection limit is 59.7 nGyair s^−1^ at 5 V bias, as indicated by the purple star surrounded by the red dashed circle. **b** Operational stability of our Cs_2_AgBiBr_6_ single-crystal X-ray detector. Testing conditions: continued 138.7 μGyair s^−1^ X-ray irradiation with constant 5 V bias, tested in ambient air without any encapsulation. Reproduced with permission from Ref. [54]. **c** Transient responses of the detector at a constant 400 V. Reproduced with permission [92]. **d** The generated voltage of multiplier tubes by scintillation light of Cs_2_NaTbCl_6_ (red line), Cs_2_NaEuCl_6_ (blue line) and (Lu,Y)_2_SiO_5_:Ce^3+^ (LYSO, black line), respectively (color online). Reproduced with permission from Ref. [93]. (Color figure online)

In 2017, Tang et al. [54] also prepared Cs_2_AgBiBr_6_ single crystals by controlling cooling rate in a solution for sensitive X-ray detectors with a low detection limit. The Cs_2_AgBiBr_6_ single crystals can directly convert X-rays into electrical signals due to the high average atomic number, high carrier drift length per unit electric field, low ionization energy and high resistivity. The optimized device exhibited high sensitivity of 105 µC/Gyair/cm^2^, low detection limit of 59.7 nGyair s^−1^ under an external bias of 5 V and demonstrated long-term operational stability, as shown in Fig. 5a, b, all of which are crucial for potential applications in X-ray security screening systems and medical diagnostics. Subsequently, Yu et al. [92] used composite films of Cs_2_AgBiBr_6_ embedded in a polymer matrix for X-ray detectors. The polymer with hydroxyl functional groups can greatly improve the uniformity of the composite films, and large area dense films can be obtained by a simple drop-casting process. X-ray detectors based on the composite films exhibit a sensitivity of 40 µC/Gyair/cm^2^ under an external bias of 400 V, as shown in Fig. 5c, and tolerate a 5% tensile/compressive strain in the composite films without performance degradation. Pixelated X-ray detectors fabricated on the same composite film can realize X-ray imaging and resolve a proof-of-concept geometric pattern. Recently, Tang et al. [93] have used lanthanide series as trivalent metals to obtain highly stable halide double perovskites (Cs_2_NaLnCl_6_, Ln=Tb or Eu) with high scintillation light yield. The crystals exhibit typical f–f transitions of lanthanide cations, while Cs_2_NaTbCl_6_ exhibits strong green photoluminescence and Cs_2_NaEuCl_6_ exhibits red photoluminescence. Under X-ray radiations, the light yield of Cs_2_NaTbCl_6_ reaches 46,600 photons MeV^−1^, much higher than that of the commercially used (Lu,Y)_2_SiO_5_:Ce^3+^ crystals (LYSO, 28,500 photons MeV^−1^), as shown in Fig. 5d. Lanthanide-based halide double perovskites open up a new route toward radiation detections and potential medical imaging.

### Photocatalyst

**Fig. 6:**
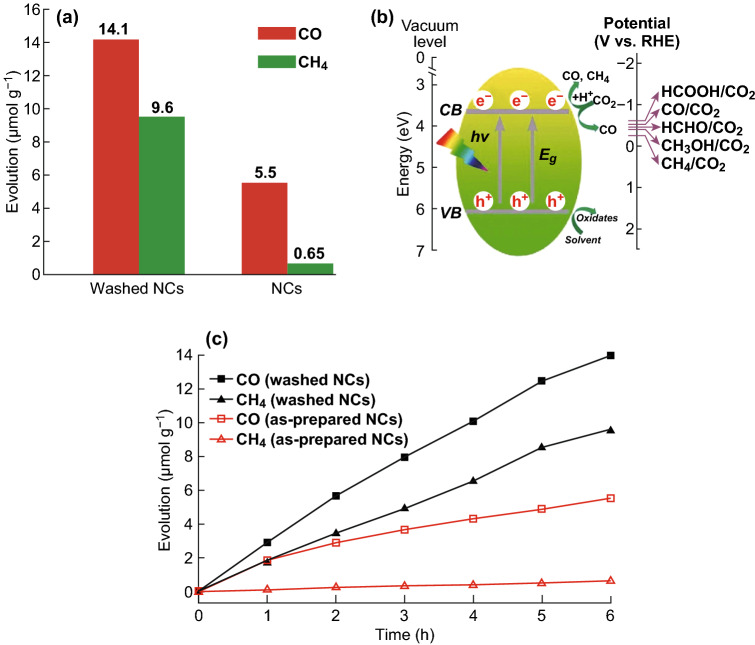
**a** Comparison of photocatalytic CO_2_ reduction performance of the as-prepared Cs_2_AgBiBr_6_ NCs and washed NCs. **b** Schematic diagram of the photoreduction of CO_2_ on the surface of Cs_2_AgBiBr_6_ NCs. **c** Time course of CO and CH_4_ evolutions over as-prepared and -washed Cs_2_AgBiBr_6_ NCs. Reproduced with permission from Ref. [56]

In 2018, Kuang et al. [56] fabricated Cs_2_AgBiBr_6_ nanocrystals (NCs) via a simple hot-injection method for photocatalytic CO_2_ reduction. The Cs_2_AgBiBr_6_ NCs can maintain their structure stability in low-polarity solutions (up to 3 weeks) and phase uniformity against 55% relative humidity for 90 days, light-soaking stability toward 70 mW cm^−2^ for 500 h or 100 °C heating for 300 h, thus declaring the impressive stability in moisture, light and temperature. Photocatalytic CO_2_ was conducted in ethyl acetate in a Pyrex glass bottle under simulated solar light (AM 1.5G, 150 mW cm^−2^) illumination. After the constant irradiation for 6 h, the pristine Cs_2_AgBiBr_6_ NCs could afford the evolution of CO and CH_4_ with 5.5 and 0.65 µmol g^−1^, respectively. Meanwhile, the electron consumption attained 16.2 µmol g^−1^, as shown in Fig. 6a. On the contrary, the washed Cs_2_AgBiBr_6_ NCs have boosted the evolution of R(CO) and R(CH_4_) to 14.1 and 9.6 µmol g^−1^, presenting a 6.5-fold enhancement in the electron consumption. A tentative mechanism for the photocatalytic CO_2_ reduction over the Cs_2_AgBiBr_6_ NCs is proposed in Fig. 6b, in which the Cs_2_AgBiBr_6_ NCs have a suitable conduction band to drive CO_2_ reduction. The time-dependent evolution of CO and CH_4_ using the as-prepared and -washed NCs by absolute ethanol as photocatalysts under light irradiation is shown in Fig. 6c, where the evolution of CO and CH_4_ rose nearly linearly with irradiation time and the washed NCs exhibited higher efficiency for CO_2_ conversion. Therefore, the novel Cs_2_AgBiBr_6_ NCs were successfully used to conduct the CO_2_ reduction reactions with high selectivity and stability, which hold great potential in the further photochemical applications.

### LEDs

Tang et al. [77] used the synthesized Bi-doped Cs_2_SnCl_6_ as blue emissive phosphors, where Bi^3+^ is the luminescent dopant. Hybrid density functional theory calculations suggest the preferred formation of [Bi_Sn_ + V_Cl_] defect complex, responsible for the optical absorption and the associated blue emission. The Bi-doped Cs_2_SnCl_6_ also shows impressive thermal and water stability due to its inorganic nature and the formation of protective BiOCl layer. A significant boost of the photoluminescence (emission peak: 455 nm, PLQY: 78.9%) was observed upon doping. This is the highest PLQY ever reported for all-inorganic lead-free perovskites and even comparable to the highest value of lead perovskites with blue emissions. Bi-doped Cs_2_SnCl_6_ showed great potential as blue phosphors, and its LED exhibited a warm white high light emission with a correlated color temperature of 4486 K and a Commission Internationale de I’Eclairage coordinate of (0.36; 0.37) when integrated with yellow phosphor. Recently, Tang et al. [94] broke the parity-forbidden transition of Cs_2_AgInCl_6_ by alloying Na^+^ cations, which leads to efficient white emission via radiative recombination of self-trapped excitons. The Bi^3+^ incorporation is believed to improve crystal perfection and promote exciton localization [95]. The optimally alloyed Cs_2_(Ag_0.60_Na_0.40_)InCl_6_ with 0.04% Bi^3+^ doping emits warm white light with (86 ± 5) % quantum efficiency and works for over 1000 h. Therefore, halide double perovskites hold great potential for display and lighting applications and merit further study to realize their full potential.

### Solar Cells

**Fig. 7:**
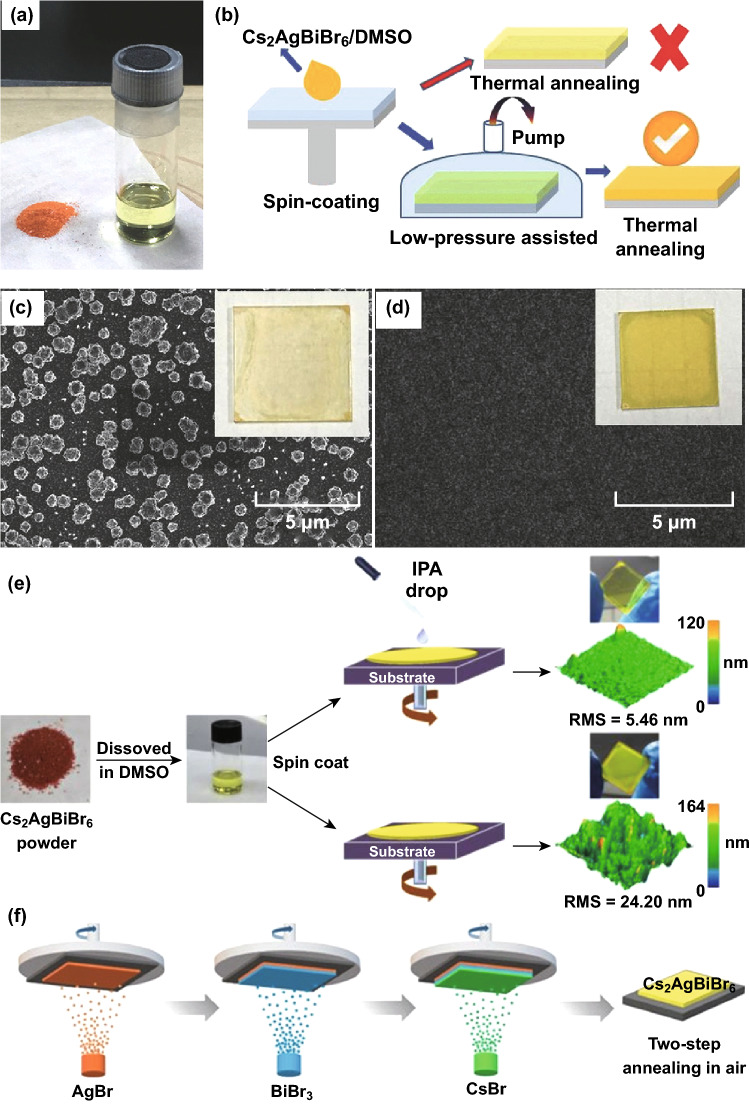
**a** Image of Cs_2_AgBiBr_6_ powder (left) and solution in DMSO (right). **b** The film fabrication process diagram. **c, d** SEM images of film obtained by traditional thermally annealed and low-pressure-assisted process, respectively, inset: film photograph, size, 25 × 25 mm^2^. Reproduced with permission from Ref. [97]. **e** Schematic illustration of the spin-coating process with and without anti-solvent dropping protocol; the morphology of the as-prepared film can be improved after IPA dropping. Reproduced with permission from Ref. [98]. **f** Scheme of sequential vapor deposition processing of Cs_2_AgBiBr_6_ double perovskite. Reproduced with permission from Ref. [100]

To date, Cs_2_AgBiBr_6_ is most applied in halide double perovskite-based solar cells [96–100]. In 2017, Bein et al. [96] fabricated Cs_2_AgBiBr_6_ films by a spin-coating method and incorporated them into solar cells for the first time. After optimized synthesis conditions, the Cs_2_AgBiBr_6_-based solar cells revealed power conversion efficiency (PCE) of 2.43% and excellent stability upon exposure without encapsulation. Inadequately, there is a thick agglomerated morphology of the Cs_2_AgBiBr_6_ films with micrometer-sized grains on the surface, and the hysteresis of the devices is serious. In 2018, Wang et al. [97] developed planar heterojunction solar cells with high-quality Cs_2_AgBiBr_6_ film by low-pressure-assisted solution processing under ambient conditions, as shown in Fig. 7a, b, borrowing Cs_2_AgBiBr_6_/SnO_2_ heterojunction from their reported detectors [91]. Orange Cs_2_AgBiBr_6_ powder was dissolved in DMSO solution to form a light yellow transparent solution and then fabricate the film using spin-coating technique, as presented in Fig. 7a. The spin-coated film was quickly moved to a low-pressure chamber pumped to 20 Pa, in which the transparent film would gradually turn to light yellow, as shown in Fig. 7b. Next, the as-prepared film was annealed and attained smooth morphology as shown in Fig. 7d. The optimized Cs_2_AgBiBr_6_ film achieved 1.44% PCE of the solar cells with P3HT hole conductor layer. On the contrary, the traditional thermal annealing method generated rough Cs_2_AgBiBr_6_ film in Fig. 7c, and the corresponding devices usually showed a poor PCE (< 0.1%). Furthermore, these devices also showed hysteresis phenomenon. Wu et al. [98] used anti-solvent dropping technology and post-annealing process to realize high-quality Cs_2_AgBiBr_6_ film with ultra-smooth morphology, microsized grains and high crystallinity (shown in Fig. 7e), which was applied in inverted planar heterojunction solar cells. The device shows PCE up to 2.23% with free hysteresis. Subsequently, Grancini et al. [99] realized hysteresis-free mesoporous double-perovskite solar cells by fine-tuning the material deposition parameters, enabling the growth of a highly uniform and compact Cs_2_AgBiBr_6_ film, and by engineering the device interfaces by screening different molecular and polymeric hole-transporting materials. Chlorobenzene was the anti-solution in the film formation before the annealing step. Recently, Liu et al. [100] utilized a sequential vapor deposition method to fabricate Cs_2_AgBiBr_6_ films for solar cells, as shown in Fig. 7f. The two-step annealing process produces films with better quality in terms of crystallization and film uniformity. The solar cells with planar device structure show an optimized PCE of 1.37%, which can be maintained at 90% after 240 h of storage under ambient condition. In addition, Ma et al. [64] incorporated their synthesized Cs_2_NaBiI_6_ into solar cells, which shows great stability and reproducibility.

It is greatly significative that the vacancy-ordered A_2_M(IV)X_6_ halide double perovskites have direct suitable bandgaps, which have been successfully applied in solar cells [74, 101–104]. In 2014, Kanatzidis et al. [74] first time introduced Cs_2_SnI_6_ as a hole-transporting material in dye-sensitized solar cells. In 2016, Cao et al. [101] discovered that B-γ-CsSnI_3_ film can spontaneously convert to an air-stable Cs_2_SnI_6_ film in air and at room temperature, which can be adopted as lead-free solar cell light absorber owing to its direct bandgap of 1.48 eV and high absorption coefficient (over 105 cm^−1^ from 1.7 eV). A planar PSC using the Cs_2_SnI_6_ film as the light absorber achieved PCE nearly 1% in air. Later on, Cao et al. [102] synthesized Cs_2_SnI_6_ powder through a modified solution process and demonstrated its application as absorbing layer in mesoporous solar cell with a configuration of FTO/ZnO compact layer/nanorods/Cs_2_SnI_6_/P_3_HT/Ag. With careful control of ZnO nanorod length and pore size to ensure high loading of the Cs_2_SnI_6_ absorber, the PCE achieved was nearly 1%. The bandgap tuning achieved by substitution of Br is anticipated to enhance the open-circuit voltage of Cs_2_SnI_6_-based solar cell. The compounds are Cs_2_SnI_6−x_Br_x_ for a range of *x* that provide the desired bandgaps from 1.3 to 2.9 eV with *x* < 3 being suitable for solar cell design. The cells show a PCE of 2.1% for the case of the *x* = 2 compound [103]. Recently, Cs_2_TiBr_6_ thin films were prepared through a facile low-temperature vapor-based method and incorporated into planar heterojunction PSCs [104]. The Cs_2_TiBr_6_ thin films exhibit favorable bandgap of 1.8 eV, long and balanced carrier diffusion lengths, suitable energy levels and superior intrinsic and environmental stability, which result in a stable solar cell with efficiency up to 3.3%.

## Conclusion and Perspective

Herein, we intend to summarize the most recent developments regarding halide double perovskite materials and the related applications. Due to distinguished stability and tunable properties, halide double perovskites have enormous potential leading to high-performance, stable and environmentally friendly optoelectronic devices for practical applications. Although significant progress has been achieved in halide double perovskites, there are still many challenges to be addressed.

Multitude of halide double perovskite materials to be existed is theoretically predicted, but a very limited number have been explored experimentally. In addition, synthesized strategies to those materials remain limited, which are mainly divided into solid-state and wet-chemical routes. The solid-state route is processed to heat anhydrous haloids as melt crystallization. Wet-chemical route includes hot injection, antisolvent recrystallization, hydrothermal methods, etc. The single crystals are classically obtained by the hydrothermal method through controlling the cooling rate from hot solution. Because a few iodine-based double perovskite materials are not easily obtained by direct combination of reactants, anion exchange and induced crystallization have been taken [51, 87]. Accordingly, we propose two aspects to make efforts: borrowing the present routes to explore the theoretical predictions and discovering viable routes to more halide double perovskite materials.

The present halide double perovskite materials exhibit excellent stability in moisture, heat and light, unlike the fashionable Pb-based perovskite materials. For instance, (MA)_2_AgSbI_6_ powder is relatively unaltered after being exposed to air for 370 days and the thermal stability can be up to 260 °C [84]. As a general rule, all-inorganic halide perovskites possess better stability [105–107]. Cs_2_AgBiBr_6_ single crystal is stable up to 430 °C, and differential thermal analysis indicates no phase transitions within this temperature range [42]. Cs_2_NaBiI_6_ exhibits superior stability against the moisture and the oxygen in the ambient air, which can be washed with water [53]. However, there is lack of proven stability under complicated environmental conditions or with long term over 10 years. In addition, the underlying cause of stability under different conditions remains covered. Hence, more attempts need to place emphasis on enhancing long-term and complicated environmental stability, as well as discovering the stability mechanisms.

Hitherto, extremely few all-inorganic and no hybrid halide double perovskite materials have been developed for optoelectronic applications. More halide double perovskite materials are desirable to be developed and widely applied. Moreover, the optoelectronic devices based on single-crystal halide double perovskite materials enjoy pleasurable performance, comparable to that of Pb-based perovskite analogs, such as Cs_2_AgBiBr_6_ single-crystal X-ray detectors [54]. However, the device performance of halide double perovskite films is still lower than that of the Pb-based perovskite analogs. The underlying causes can be ascribed to the underdeveloped electronic structures, material properties, film qualities of halide double perovskite materials and device architectures. It is difficult to develop a synthetic route to obtain uniform thin films of the correct phase and composition. Typically, Cs_2_AgBiBr_6_ [96], Cs_2_SnI_6-x_Br_x_ [103] and Cs_2_TiBr_6_ [104] absorbers in PSCs have achieved optimized efficiency of 2.4, 2.1 and 3.3%, respectively, far below that of the Pb-based perovskite analogs. To enhance Cs_2_AgBiBr_6_-film quality, anti-solvent dropping and low-pressure-assisted solution methods have been adopted [97, 98]. Interface engineering of device architectures was taken to achieve hysteresis-free Cs_2_AgBiBr_6_-based PSCs [99]. However, the performance is still unsatisfactory because Cs_2_AgBiBr_6_ has a relatively large indirect bandgap. Various strategies can be attempted to engineer the bandgap and modify the device structures to improve the device performance, such as chemical doping and alloying approaches. Meanwhile, chemical doping and alloying approaches are expected to bring original effects for applications. As an example, Bi-doped Cs_2_SnCl_6_ can be applied as blue emissive phosphors, where Bi^3+^ is the luminescent dopant [77].

In summary, we are very much optimistic that the current astonishing achievements will encourage more researchers to overcome the above challenges in the future.
